# Glutamine/Glutamate Metabolism Studied with Magnetic Resonance Spectroscopic Imaging for the Characterization of Adrenal Nodules and Masses

**DOI:** 10.1155/2013/835385

**Published:** 2013-10-02

**Authors:** Suzan M. Goldman, Thiago F. Nunes, Homero J. F. Melo, Claudio Dalavia, Denis Szejnfeld, Claudio Kater, Cassio Andreoni, Jacob Szejnfeld, Sergio A. Ajzen

**Affiliations:** ^1^Department of Diagnostic Imaging, Federal University of São Paulo, Napoleão de Barros 800, Vila Clementino, 04024-002 São Paulo, SP, Brazil; ^2^Department of Endocrinology, Federal University of São Paulo, Pedro de Toledo 650, 2nd Floor, Vila Clementino, 04024-002 São Paulo, SP, Brazil; ^3^Department of Urology, Federal University of São Paulo, Napoleão de Barros 715, 4th Floor, Vila Clementino, 04024-002 São Paulo, SP, Brazil

## Abstract

*Purpose*. To assess glutamine/glutamate (Glx) and lactate (Lac) metabolism using proton magnetic resonance spectroscopic imaging (1H-MRS) in order to differentiate between adrenal gland nodules and masses (adenomas, pheochromocytomas, carcinomas, and metastases). *Materials and Methods*. Institutional review board approval and informed consent were obtained. A total of 130 patients (47 men) with 132 adrenal nodules/masses were prospectively assessed (54 ± 14.8 years). A multivoxel system was used with a two-dimensional point-resolved spectroscopy/chemical-shift imaging sequence. Spectroscopic data were interpreted by visual inspection and peak amplitudes of lipids (Lip), choline (Cho), creatine (Cr), Lac, and Glx. Lac/Cr and Glx/Cr were calculated. Glx/Cr was assessed in relation to lesion size. *Results*. Statistically significant differences were observed in Glx/Cr results between adenomas and pheochromocytomas (*P* < 0.05), however, with a low positive predictive value (PPV). Glx levels were directly proportional to lesion size in carcinomas. A cutoff point of 1.44 was established for the differentiation between carcinomas larger versus smaller than 4 cm, with 75% sensitivity, 100% specificity, 100% PPV, and 80% accuracy. Lac/Cr results showed no differences across lesions. A cutoff point of −6.5 for Lac/Cr was established for carcinoma diagnosis. *Conclusion*. Glx levels are directly proportional to lesion size in carcinomas. A cutoff point of −6.5 Lac/Cr differentiates carcinomas from noncarcinomas.

## 1. Introduction

Proton magnetic resonance spectroscopic imaging (^1^H MRS) has become increasingly consolidated as a noninvasive and risk-free procedure for disease followup [1]. ^1^H MRS assessment is based on metabolite levels present in a certain volume as shown on magnetic resonance imaging (MRI) scans.

In a previous study, we reported our initial experience in distinguishing adrenal adenomas, pheochromocytomas, adrenocortical carcinomas, and metastases based on ^1^H MRS data, using histological/computed tomographic findings and followup data as [2]. Since then, several protocol improvements have been implemented in our institution, which have allowed the inclusion of new metabolites in the analysis, namely, lactate (Lac) and the glutamine/glutamate complex (Glx) [3].

Glucose and glycogen levels are low in tumoral cells. Therefore, glycolysis regulation in these structure occurs via alternative carbohydrates, especially glutamine. The degradation of to lactate has been referred to as glutaminolysis [4].

There is no consensus in the literature regarding the association between high or low concentrations of glutamine and tumor growth potential [5–7]. Nevertheless, similarly to glycolysis, the main role of glutaminolysis is to generate energy, via the production of glutamate, citrate, and aspartate. One advantage associated with glutaminolysis is that it maintains glutamin levels constantly high in cancerous tissues and solid tumors [4].

Compared with normal cells, tumors use almost all their ability to degrade glucose, independent of the amount of oxygen present. However, this consumption under anaerobic conditions can be up to 20 times higher than in aerobic conditions [8]. In addition, several researchers have demonstrated that lactate stimulates angiogenesis [9]. 

^1^H MRSI in vivo is not able to distinguish between glutamin and glutamate. As a result, total Glx data are usually assessed, considering a peak between 2.1 and 2.5 ppm [1, 10–17]. Notwithstanding, to the authors' knowledge, no study so far has assessed glutamine/glutamate metabolism characteristics in the adrenal gland and correlated these findings with the possibility to differentiate between nodules and masses.

Therefore, the objective of this study was to assess Glx and Lac metabolism using ^1^H MRS in order to differentiate between adrenal nodules and masses (adenomas, pheochromocytomas, carcinomas, and metastases).

## 2. Materials and Methods

The present study was approved by the appropriate Research Ethics Committee. Informed consent forms were signed by all patients included or their guardians.

### 2.1. Patients

A total of 130 patients (47 males) presenting with adrenal nodules or masses were prospectively assessed. All patients had been previously examined with a dedicated adrenal CT and/or a MR protocol. Of the 130 patients, 128 presented nodules or masses either on the right or left adrenal gland only, and two presented bilateral masses or nodules, totaling 132 nodules. All patients underwent ^1^H MRSI at the Department of Diagnostic Imaging of our institution. Data collection took place between January 2004 and May 2011.

The following inclusion criteria were taken into consideration: (1) presenting with adrenal nodule/mass with a diameter >1.0 cm and previous dedicated adrenal CT or MRI exam; (2) histopathological confirmation by biopsy or surgery in cases of pheochromocytomas, carcinomas, functional adenomas, or nonspecific lesions; (3) lesion stability for over 12 months based on CT or MRI findings in patients with adenomas. Patients on chemotherapy protocols or with previous adrenal biopsy or surgery, as well as those in which it was not possible to schedule the ^1^H MRSI sequence, were not included in the sample.

Exclusion criteria were presence of lesion(s) with a diameter >1.0 cm but no voxel eligible for analysis (consisting of 100% tumoral tissue), and presence of adrenal nodule or mass with a diameter around 1.0 cm but presenting surface contaminants.

Five patients were excluded as a result of poor band positioning, a low signal-to-noise ratio, and absence of voxels eligible for analysis. As a result, a total of 125 patients (44 males) met = all study criteria and were therefore included in the final analysis. Mean age in this group was 54 ± 14.8 years (range: 13–87), and all presented with an adrenal lesion larger than 1.0 cm (mean ± standard deviation: 4.1 ± 2.68 cm; range: 1.19–14.35 cm). A total of 127 masses were diagnosed and classified as adenomas (84), carcinomas (10), pheochromocytomas (28), and metastases (5).

### 2.2. ^1^H MRSI Protocol

Patients were examined after a four-hour fast and 10 minutes after the intravenous administration of an antispasmodic drug (Buscopan). At this occasion, patients were also asked to answer a questionnaire on MRI contraindications.

Patients were positioned feet first into the MRI system, with their arms resting alongside their bodies, and lying down on their backs on the spine coil. Once the patient was adequately positioned, the phased-array spine coil was used. 

MRI examinations were conducted using either a 1.5 T Magnetom Sonata scanner (gradient 43 mT/m; Siemens Medical Systems, Erlangen, Germany), a 1.5 T Magnetom Espree scanner (33 mT/m; Siemens Medical Systems) (*n* = 123), or a 3.0 T Magnetom Verio scanner (Siemens Medical Systems) (*n* = 4). 

MRI imaging was performed at the level of the adrenal mass and consisted of T2-weighted half-Fourier acquisition single shot turbo spin echo (HASTE) sequences and T1-weighted chemical shift imaging (CSI). 

HASTE sequences were performed in three orthogonal planes to allow three-dimensional (3D) mass location and thus planning of the shimming and ^1^H MRSI protocol, as described by Faria et al. [2].

Subsequently, spectroscopic imaging data were obtained using a multivoxel system in the selection of the volume of interest and a hybrid two-dimensional point resolved spectroscopy (PRESS)/CSI sequence, commercially available from Siemens Medical Systems. During examination we reinforce for the patient to breathe smoothly. This protocol was adopted so as to minimize any possible artifacts from periadrenal structures.

**Figure 1:**
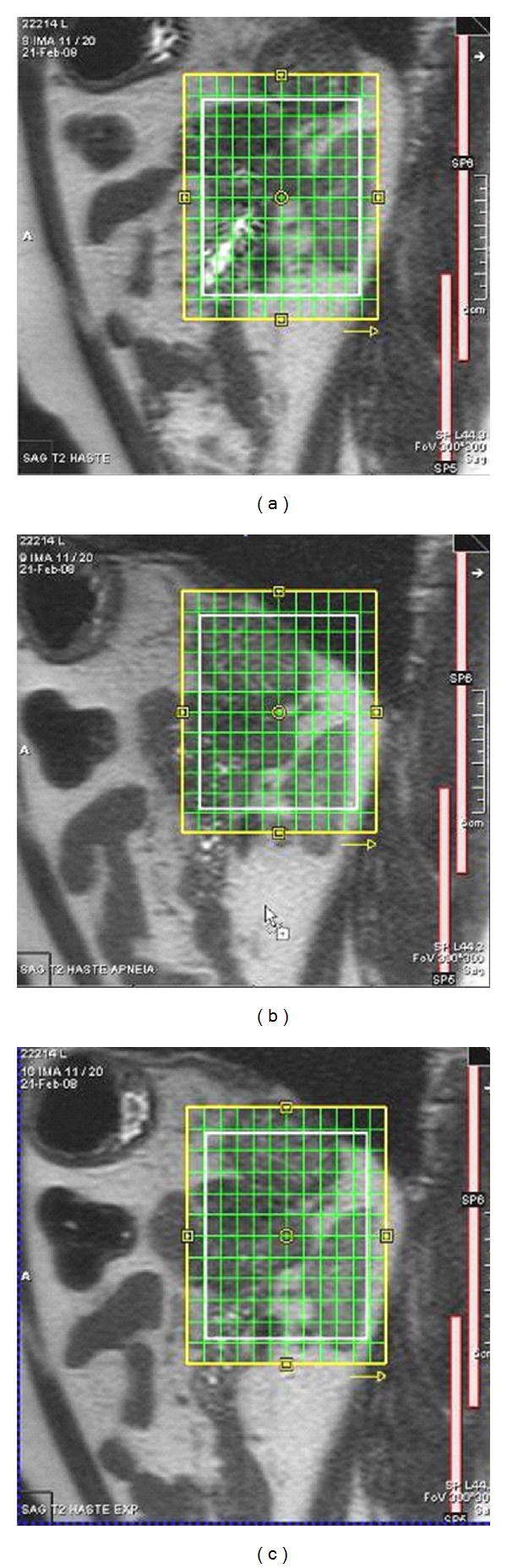
T2 HASTE sagittal (left adrenal adenoma): (a) maximun inspiration; (b) maximun expiration; (c) free breathing.

**Figure 2:**
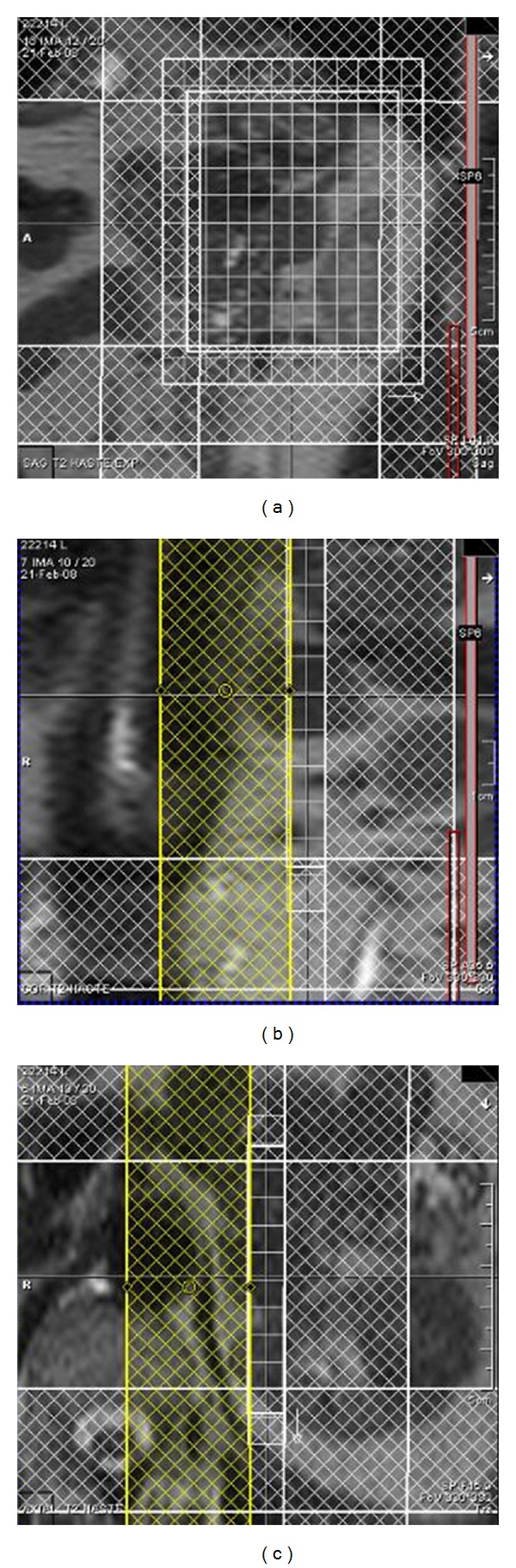
Final positioning of ^1^H MRSI scan: (a) T2 HASTE in coronal orientation in the center of the left adrenal adenoma; (b) T2 HASTE in axial orientation in the center of the left adrenal adenoma; (c) T2 HASTE in sagittal orientation including all adrenal glands, lesions, and the breathing interval calculated as in Figures 1(a), 1(b), and 1(c).

^1^H MRSI data were obtained using T2-weighted HASTE sequences, in two stages: (1) sagittal images only, at maximum inspiration, maximum expiration, and free breathing, carefully positioning the multivoxel volume of interest grid in the center of the lesion, based on all three sagittal sequences (Figure 1), to include as much of the lesion area as possible (or the whole lesion and part of adjacent fat tissue, if possible), and (2) three orthogonal planes at expiration, in order to determine voxel size and to activate only the coil located nearest to the mass/nodule under analysis, as well as to adequately position outer volume saturation bands (Figure 2).

Six 30 mm wide saturation bands were used, positioned around the adrenal gland, thus minimizing possible field heterogeneity effects caused by magnetic interference of air present in the lung parenchyma, bone structures, periadrenal fat, and fluids present in the biliary tree and kidneys.

Adrenal ^1^H MRSI images were obtained using a voxel size of 0.56–3.38 cm^3^, a field of view of 100–150 cm, 4–8 excitations, repetition time of 1500 ms, an echo time of 135 ms, and a delta factor of −3.4, *k*-space elliptical. Image acquisition lasted between 7 minutes and 11 minutes 45 seconds. Water suppression was used during spectroscopic examination so that the lipids present in the gland would be measured on the images (MEGA technique) [17].

### 2.3. Spectroscopic Data Analysis

All spectroscopic findings were analyzed by two investigators (Homero J. F. Melo and Suzan M. Goldman) until consensus was reached. Images were processed using a Leonardo workstation (Siemens Medical Systems). A 1000 Hz Gaussian line-broadening filter was used, and priority was given to the transformation of Fourier data into two spatial directions, using a Hamming filter.

The ^1^H MRSI matrix was adjusted to the three orthogonal planes obtained during image acquisition, and the most appropriate voxels were selected for analysis. Voxels located in the adjacent fat tissue (not including lesion tissue) were disconsidered. Spectroscopic analysis was conducted in left-right, craniocaudal direction. Following voxel selection, the abscissa was enlarged so that an adequate ppm amplitude (0.5–8.5) could be used to phase correct water peak in relation to other metabolites. Subsequently, the ppm range was reduced (0.5–4.7) again in order to increase spectral resolution. 

All spectroscopic data were interpreted by means of visual inspection and by measuring peak amplitudes of the metabolites of interest, namely, creatine (Cr), lactate (Lac), glutamine, and glutamate (Glx) (2.1–2.5 ppm).

In addition, Glx and Lac were measured in the 2.1–2.5 ppm range and 1.33 ppm, respectively, and their relationship with Cr (Glx/Cr and Lac/Cr) was calculated. The association between Glx-Cr ratios and lesion size was also assessed.

### 2.4. Statistical Analysis

Data were analyzed using Excel and BioEstat 4.0 software for the metabolic characterization of the four types of masses or nodules analyzed, namely, adenomas, carcinomas, pheochromocytomas, and metastases. Central tendency (mean and mode) and dispersion values (standard deviation and range) were calculated for each group. Student's *t*-test for paired samples was used to compare mean metabolic ratios obtained in the groups, and the chi-square test was used to correlate differences in the relationships, followed by Yates' correction or Fisher's exact test for cell values below five.

The association between Glx-Cr ratios and nodule size was assessed using Pearson's linear correlation. Also, receiver operating characteristic (ROC) curves were used to determine cutoff points, sensitivity, specificity, positive predictive value (PPV), and accuracy were calculated for the cutoff points established. Significance was set at *P* ≤ 0.05. 

## 3. Results

**Figure 3:**
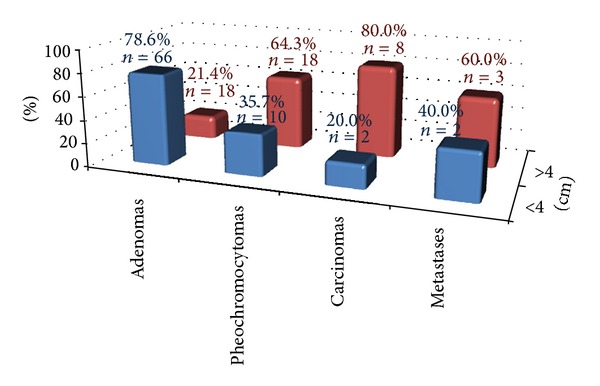
Number of masses and nodules according to nodule size.

^1^H MRSI was successfully performed in 127 (96.2%) of the 132 adrenal masses and nodules assessed.  Figure 3 shows the types and sizes of lesions identified.

**Figure 4:**
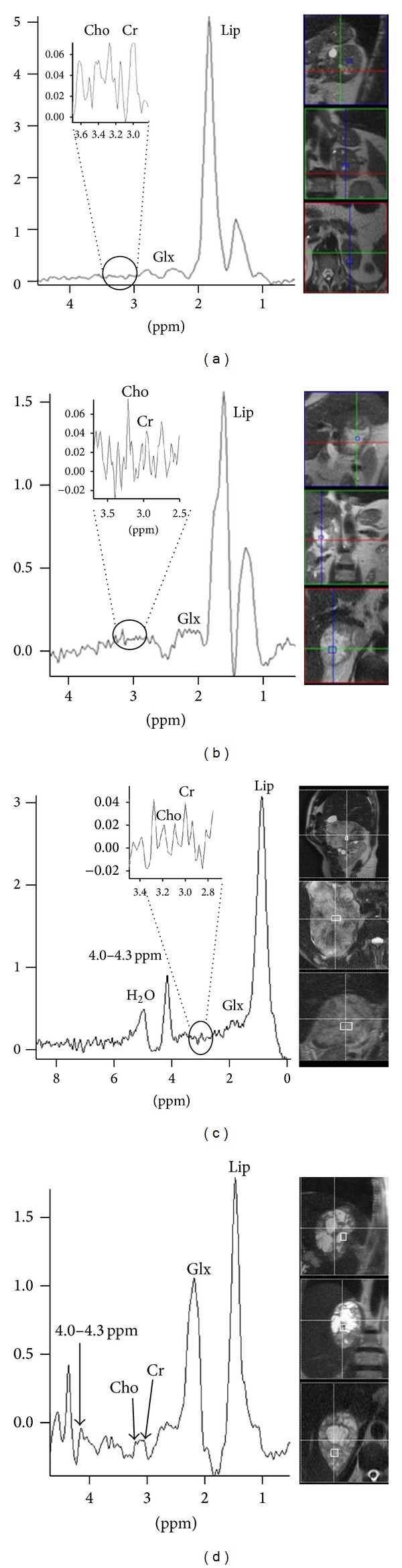
Spectroscopic findings: (a) adenomas; (b) metastases; (c) pheochromocytomas; (d) carcinomas. Cho, Cr, Lip, Glx, Lac, and H_2_O peaks are indicated.

Figure 4 shows spectroscopic findings of the nodules assessed.

Table 1 shows the results obtained for Glx/Cr and Lac/Cr ratios in the lesions assessed. 

**Figure 5:**
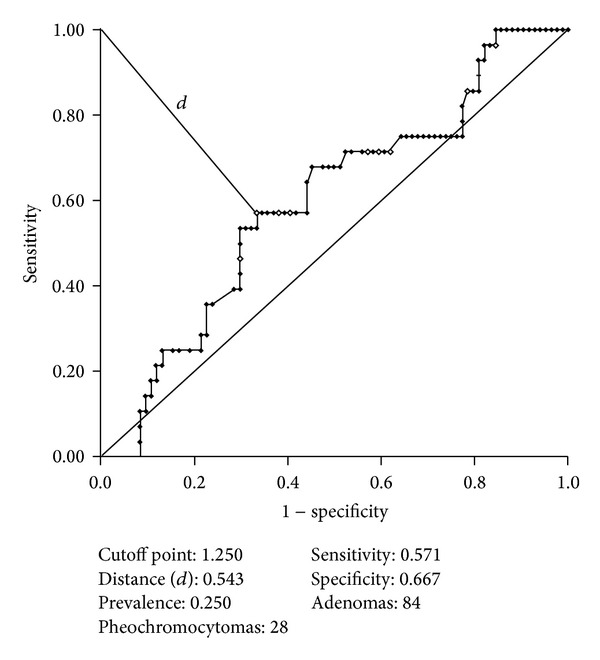
Receiver operating characteristic curve calculated for Glx/Cr between adenomas and pheochromocytomas.

Statistically significant differences were observed in Glx/Cr results between adenomas and pheochromocytomas (*P* < 0.05), however, with a low PPV. The ROC curve calculated did not reveal a cutoff point that could allow adequate differentiation between these two types of lesions (57.1% sensitivity, 66.7% specificity, 36.37% PPV, and a 64.30% accuracy for a Glx-Cr ratio ≥1.25) (Figure 5). In the other groups, it was not possible to establish a cutoff point based on Glx/Cr results. 

**Figure 6:**
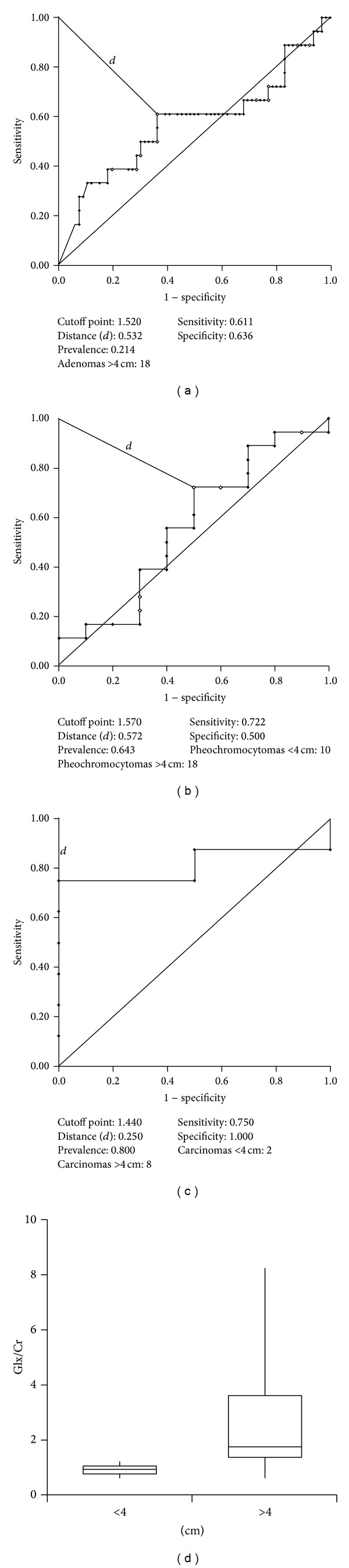
Receiver operating characteristic curves calculated for Glx/Cr according to lesion size: (a) adenomas, (b) pheochromocytomas, and (c) carcinomas. (d) Box plot showing variations in carcinoma lesion size.

Glx levels were found to be directly proportional to lesion size in carcinomas.  Figure 6 shows ROC curves calculated for Glx/Cr in carcinomas, pheochromocytomas, and adenomas in relation to lesion size (masses or nodules larger versus smaller than 4 cm). Statistically significant findings were observed for carcinomas (*P* < 0.05), but not for the other two lesions. A cutoff point of 1.44 was established for the differentiation between carcinomas larger versus smaller than 4 cm (Figure 6(c)), with 75% sensitivity, 100% specificity, 100% PPV, and 80% accuracy. Metastases were not included in this analysis because of the small number of specimens available. 

Lac/Cr was also calculated for the different types of lesions in an attempt to define a cutoff point for differentiation. However, no significant differences were found in the comparison of adenomas versus nonadenomas (*P* = 0.063; 81% sensitivity, 63% specificity). Conversely, we did observe a tendency toward increased Lac concentrations in nonadenomas.

**Figure 7:**
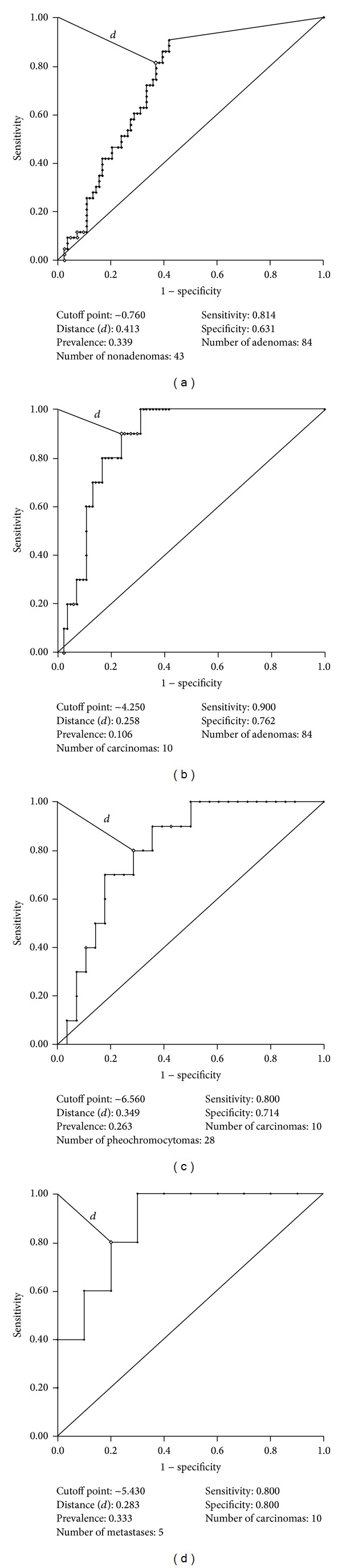
Receiver operating characteristic curves calculated for Lac/Cr between the following groups: (a) adenomas versus nonadenomas; (b) carcinomas versus adenomas; (c) carcinomas versus pheochromocytomas; and (d) metastases versus carcinomas.

The ROC curve calculated for differentiating between adenomas and nonadenomas shown in Figure 7(a) yielded a cutoff point of −0.76, with 81.4% sensitivity, 63.1% specificity, 53.8% PPV, and 69.3% accuracy. Student's *t* test did not reveal statistically significant differences (*P* = 0.063), despite a trend toward differentiation. The ROC curve for adenomas and carcinomas (Figure 7(b)) determined a cutoff point of −4.25, with 90% sensitivity, 76.2% specificity, 30.96% PVV, and 77.66% accuracy. Conversely, the ROC curve for the differentiation between pheochromocytomas and carcinomas, shown in Figure 7(c), yielded a cutoff point of −6.56, with 80% sensitivity, 71.4% specificity, 49.95% PPV, and 73.66% accuracy. Finally, the ROC curve for the differentiation between metastases and carcinomas (Figure 7(d)) resulted in a cutoff point of −5.34, with 80% sensitivity, 80% specificity, 66.63% PPV, and 80% accuracy.

**Figure 8:**
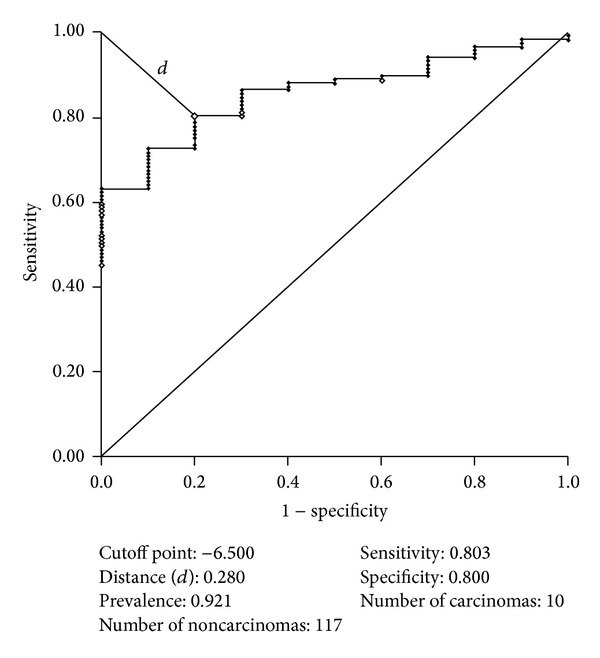
Receiver operating characteristic curve calculated for Lac/Cr between carcinomas and noncarcinomas.

Because all comparisons in carcinoma lesions yielded statistically significant differences, a cutoff point was calculated with the ROC curve for the differentiation between carcinomas and noncarcinomas (*P* < 0.05) (Figure 8), namely −6.5.

## 4. Discussion

The present study tested the effectiveness of an ^1^H MRSI protocol in differentiating between adrenal masses and nodules (adenomas, pheochromocytomas, carcinomas, and metastases) based on the previously tested Lac/Cr and Glx/Cr ratios.

The first studies using ^1^H MRSI to assess the adrenal gland in vivo were aimed at locating the region of interest using spectroscopic images [18]. Although those early results were satisfactory, some limitations of the methodology deserve to be mentioned: the impossibility to build spectral graphs, mass/nodule visualization in one single orthogonal plane only, low magnetic field power, and visualization of water and fat tissues only. In 2009, Kim et al. proposed the use of respiratory-triggered proton single-voxel MR spectroscopy in vivo for adrenal gland investigation [19]. Again, early results were promising, but the limitations inherent to single-voxel MRI weakened overall findings and conclusions.

In an attempt to overcome such limitations, two-dimensional multivoxel systems started to be used to locate and select the spectroscopic region of interest, starting from the sagittal plane of the adrenal gland [2]. In multivoxel image acquisition, the exact location of the lesion becomes less important, as more relevant information is provided regarding the tumor's spatial extent and metabolic heterogeneity [20]. 

The present study describes some changes made to the ^1^H MRSI acquisition and analysis protocol originally proposed [2]. Among image acquisition improvements, it is possible to mention free multivoxel angulation, a better adjustment of the field of view to the mass or nodule under analysis and a better signal-to-noise ratio, in addition to the two-dimensional sequence adopted. Conversely, the use of three sagittal planes (maximum inspiration, maximum expiration, and free breathing), with a limited shimming area, was maintained, as the authors believe that this strategy compensates the absence of respiratory monitoring in multivoxel sequences. 

Whole-body scanners with a magnetic field strength of 3 T are becoming more and more available to the scientific and clinical communities [21]. The advantages of spectroscopy at higher field strengths are mainly an increased signal/noise ratio (SNR), improved spectral, spatial, and temporal resolution, including detection of resonances obscured at 1.5 T (such as amino acids), and enhancement of J-coupled spectral resolution [22]. 

With regard to image analysis, changes made to the protocol reflect the technological improvements incorporated by the spectroscopic systems themselves (Siemens Medical Systems). In particular, the possibility to use different scales, from higher (0.5–8.5 ppm) to lower (0.5–4.7 ppm) amplitude values, allowed to better adjust the base line, taking into consideration peak amplitude measurements calculated for water and other metabolites in the automatic and manual determination of spectral phase and frequency. Whenever necessary, amplitude values were lowered to 2.5–3.5 ppm to improve the visualization of Cho and Cr peaks, and voxel size was increased or decreased according to lesion size. All these strategies contributed to a faster and safer selection of eligible voxels.

Moreover, adrenal oncocytoma does not follow the rule of four: (a) 4% diagnosed on CT, (b) 4% of these tumors are pheochromocytoma or adrenocortical carcinoma, (c) ≥4 cm in size is an indication for surgery, and (d) 4 years of followup is required [23]. 

High Glx and Lac peaks were observed, and therefore their relationships with Cr were assessed. Both metabolites showed a great variance across the different lesions analyzed. The analysis did not reveal statistically significant associations between Glx/Cr and different types of masses/nodules, but rather with lesion size: a cutoff point of 1.44 was established for the differentiation between carcinomas larger versus smaller than 4 cm; no differences were observed in other lesions. Although the number of carcinomas included in our analysis was small (only 10 lesions), our results gain importance if we take into consideration the very low prevalence of this type of tumor in the general population [5]. 

Conversely, Lac/Cr did not show statistically significant differences in the differentiation between adenomas and nonadenomas (*P* = 0.063). Notwithstanding, the following cutoff points could be established: adenomas versus carcinomas (−4.25), pheochromocytomas versus carcinomas (−6.56), and carcinomas versus metastases (−5.34) (*P* < 0.05). A cutoff point of −6.5 for Lac/Cr was established for the diagnosis of carcinomas. These findings therefore add to the existing literature, and especially to our previous study [2] and contribute to clinical practice by improving our capacity to accurately diagnose and differentiate between these types of tumor.

Some limitations of the present study deserve to be mentioned. First of all, as a result of the strict inclusion criteria adopted, our sample included a very small number of metastases (only five), preventing the inclusion of this category in the statistical analyses. Another limiting factor is the reduced number of patients using a 3.0 T scanner, rendering impossible comparisons between the curves generated by different fields. Finally, our two examiners had experience in spectroscopy, and maybe readings made by less experienced examiners could yield different results. This potential limitation is important because spectroscopic analysis of adrenal masses is still gaining space, especially if compared with areas in which it is highly consolidated, such as prostate cancer.

Future studies are warranted to investigate the possibility of differentiation between functional and nonfunctional adenomas, still a major difficulty in the management of these patients, as well as to compare in vivo and in vitro spectroscopic findings with the aim of identifying metabolites present in different masses.

In sum, the ^1^H MRSI sequences adopted in the present study proved effective for the differentiation between adrenal masses and nodules and data analysis was effective and reliable. Other studies are currently underway that focus on lipid concentrations as observed on spectral graphs compared with laboratory tests.

Our main findings can be summarized as follows: Glx levels are directly proportional to lesion size in carcinomas, and Lac/Cr allows to differentiate between carcinomas and noncarcinomas.

## Figures and Tables

**Table 1:** Descriptive analysis of lesions with regard to Glx/Cr and Lac/Cr ratios*.

	Glx/Cr			Lac/Cr		
	Mean ± SD	Median	Range	Mean ± SD	Median	Range
Adenomas	2.13 ± 1.88	1.69	0–8.77	−3.39 ± 7.12	0	−38.84–1.72
Pheochromocytomas	1.37 ± 1.20	1.13	−1–4.05	−4.64 ± 5.88	−2.5	−22.9–0.93
Carcinomas	2.65 ± 2.82	1.55	0.56–8.2	−10.8 ± 6.34	−9.04	−21.9–−2.56
Metastases	1.95 ± 1.44	1.70	0.12–4.1	−3.62 ± 2.98	−4.23	−7.3–0

Glx/Cr: glutamine/glutamate-creatine ratio; Lac/Cr: lactate-creatine ratio; SD: standard deviation.

*All comparisons resulted nonsignificant (*P* > 0.01).
